# Partial Occlusion of the Vagina and an Almost Imperforate Hymen, in Case of a Lunatic, with Recovery after Operation

**Published:** 1876-04

**Authors:** Thomas H. Kenan

**Affiliations:** 2d Assistant Physician, Georgia Lunatic Asylum


### PARTIAL OCCLUSION OF THE VAGINA AND AN ALMOST IMPERFORATE HYMEN, IN CASE OF A LUNATIC, WITH RECOVERY AFTER OPERATION.

By THOMAS H. KENAN, 2d Assistant Physician, Georgia Lunatic Asylum.

On October 12th, 1874, Miss A. ; age 42 ; lunatic ; duration of insanity 3 or 4 years ; health very feeble for years ; was found to be more excited, and in great pain, with desire to micturate often at each menstrual period. Upon examination, there was found to all appearance, no vagina ; but upon close inspection there was found a band of flesh about the one sixth of an inch wide leading across the vagina from near "meatus urinarius" to "navicular fosa," leaving two openings, each the size of a small goose quill, Failing to introduce the finger, a silver catheter was passed into one and out of the other opening leaving band on the stretch over catheter. The band was cut, and after some little effort, the index finger was carried in, and an almost imperforate hymen found, which was with some slight difficulty broken down. Uterus normal in size, etc.; there was very little hemorrhage ; ordered an injection of slippery elm in warm water, daily, for a few days ; was kept in bed for a day, when she got up and expressed herself as feeling very well, and at the following regular menstrual period she had no trouble; was discharged restored December 12th, 1874. At the time of writing, April 20th, 1875, she is working for her support: cooks, washes, irons, and does other household duties for a family, and wrote a letter saying she was able to do as much work in a day as any ordinary female employee.

